# Vertical parasagittal hemispherotomy for Sturge–Weber syndrome in early infancy: case report and literature review

**DOI:** 10.1186/s40064-016-3096-2

**Published:** 2016-08-30

**Authors:** Xiangyu Liu, Taisuke Otsuki, Akio Takahashi, Takanobu Kaido

**Affiliations:** 1 Department of Neurosurgery, Jinling Hospital, School of Medicine, Nanjing University, 305 East Zhongshan Road, Nanjing, 210002 Jiangsu Province People’s Republic of China; 2 Department of Neurosurgery, National Center of Neurology and Psychiatry, 4-1-1 Ogawahigashi-cho, Kodaira, Tokyo 187-8551 Japan; 3 Department of Neurosurgery, National Hospital Organization Nara Medical Center, Shichijo 2-789, Nara, 630-8053 Japan

**Keywords:** Vertical parasagittal hemispherotomy, Sturge–Weber syndrome, Epilepsy surgery, Seizure

## Abstract

**Introduction:**

The authors here present a rare case of a 3-month-old infant with unilateral Sturge-Weber syndrome (SWS) who had excellent seizure control and no aggravation of previous existed neurological deficits after vertical parasagittal hemispherotomy (VPH). To our knowledge, this patient with SWS was the youngest one who received VPH.

**Case description:**

The use of VPH results in a successful treatment of intractable epilepsy in a patient with seizure onset in early infancy. At follow-up, the patient’s neurodevelopmental status has been improved since the surgery.

**Discussion:**

It is generally accepted that early-onset seizures in children with SWS are associated with worse neurological and developmental outcomes. However, when surgical treatment should be considered and how it should be performed remain a longstanding controversy. We promote early surgery in children with SWS and early-onset epilepsy.

**Conclusion:**

We suggest that VPH may be a useful adjuvant in the management of SWS with refractory epilepsy in early infancy and this procedure carries low neurological risk.

## Background

Sturge–Weber syndrome (SWS) is a rare congenital neurocutaneous disorder characterized by facial port wine stains and associated intracranial leptomeningeal angiomatosis. Seizures are the most common manifestation of the disease and two thirds of patients with SWS present with seizures during the first year of life (Bachur and Comi 2013; Jagtap et al. 2013; Alkonyi et al. 2011). It is well established that early-onset seizures are more difficult to control and may lead to neurological and developmental deterioration. The appropriate treatment depends on the available natural history and early surgery is advocated to attain seizure control in those with severe medically intractable epilepsy (Schramm et al. 2012; Honda et al. 2013; Kramer et al. 2000). Introduced by Delalande in 1992, vertical parasagittal hemispherotomy (VPH) has been described as an effective surgical technique for hemispheric disconnection. This technique allows complete disconnection of the hemisphere through a cortical window with good results of seizure control and gets the utmost degree of vessel preservation within the disconnected hemisphere to reduce the risk of ischemic cerebral edema (Delalande et al. 2007; Delalande and Dorfmuller 2008). Although VPH is a newly developed hemispherotomy technique, it can be a useful adjuvant in the management of epilepsy for SWS patients in early infancy and may help preserve neurological function by preventing progressive neurological deterioration and intellectual impairment.

## Clinical presentation

### History

**Fig. 1:**
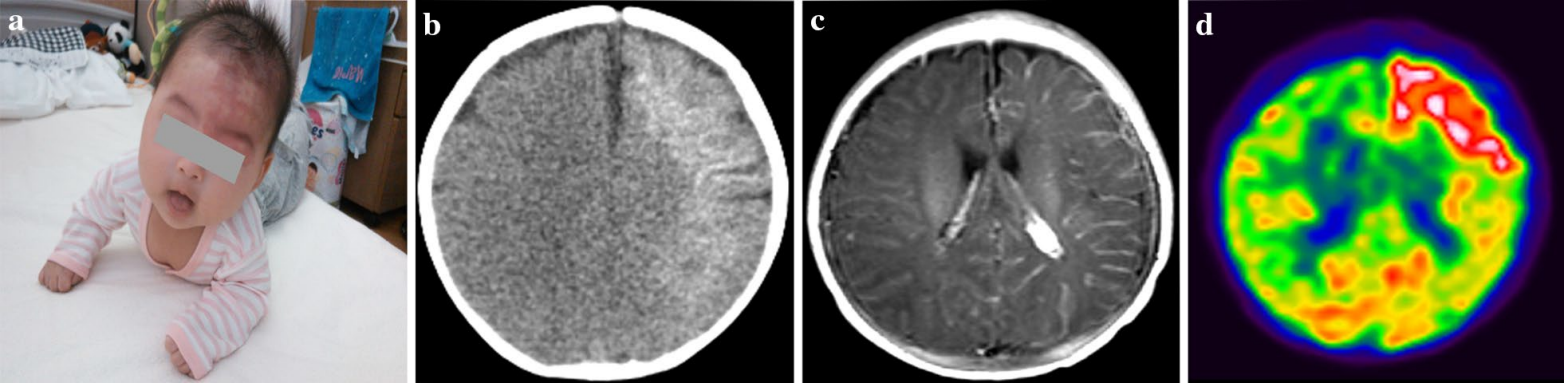
**a** Facial port-wine stain affecting the facial skin; **b** CT scan revealing cortical calcification in the cerebral hemisphere; **c** MR imaging demonstrating leptomeningeal venous angioma and enlargement of the choroid plexus; **d** An immediate postictal FDG-PET scan showing significant intense hypermetabolism in the left frontal lobe

This 3-month-old infant was noticed at birth have an unilateral congenital nevus flammeus (port-wine stain) (Fig. 1a). She did not show any neurologic manifestations until 51 days after birth when she developed her first seizure. She had no family history of seizures or epilepsy, and her development was age-appropriate. Her brother and mother developed febrile convulsion during their infancy. The diagnosis of SWS was established by the presence of the facial nevus flammeus and seizures. The patient’s legal parent gave consent to publish this case report and any accompanying images.

**Fig. 2:**
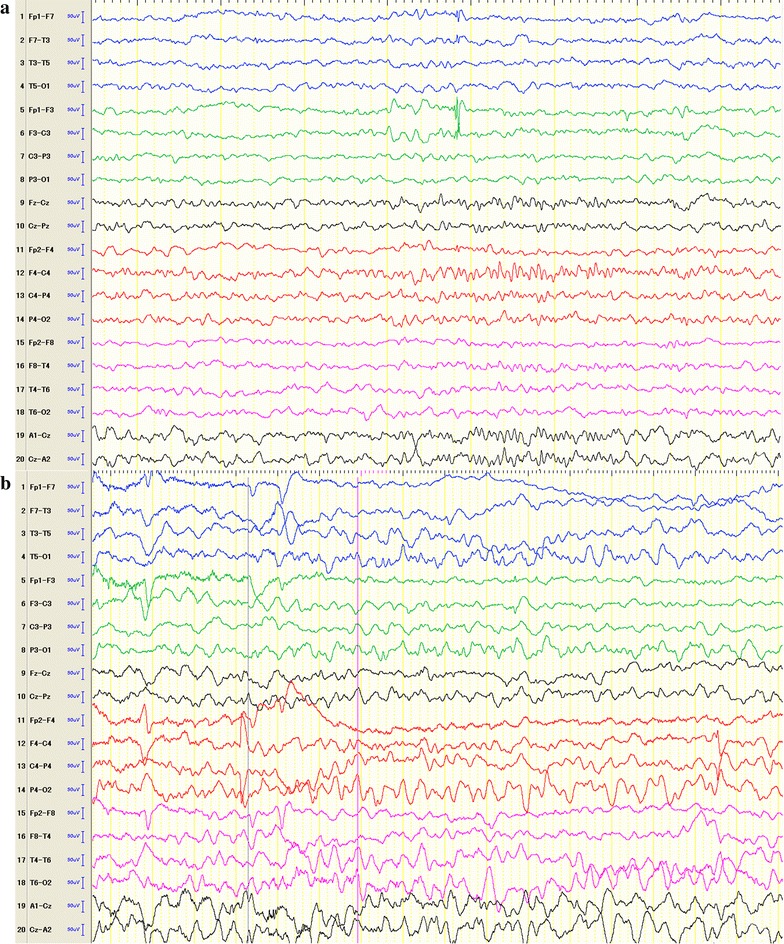
**a** Interictal scalp EEG showing markedly asymmetry with spikes observed on F3&F7; **b** Ictal scalp EEG revealing changing of the background activities and bilateral rhythmic slow waves with no localized value

Computerised tomography (CT) scan revealed cortical calcification in the left cerebral hemisphere (Fig. 1b). Magnetic Resonance (MR) imaging demonstrated unilateral cerebral atrophy and leptomeningeal venous angioma (Fig. 1c). Initially, her seizures were well controlled by the combination therapy with phenobarbital and clonazepam. However, the patient developed mild right hemiparesis and tonic seizures with focal features at a frequency of 4–5 times per day which were refractory to medication. She was admitted to our hospital and underwent a thorough general physical and neurological examination at 3 months of age. The interictal scalp electroencephalography (EEG) (Fig. 2a) showed obvious asymmetry with spikes detected on left frontal and anterotemporal area, and the ictal scalp EEG revealed changing of the background activities and bilateral rhythmic slow waves with no localized value (Fig. 2b). Immediate postictal evaluation of positron emission tomography (PET) demonstrated significant intense hypermetabolism in the left frontal lobe (Fig. 1d).

### Operation and postoperative following up

Preoperatively, central venous catheterization for blood transfusion was placed in this infant. Intraoperative blood loss was estimated and blood component transfusion including packed red blood cells and fresh frozen plasma (FFP) was used during surgery. As our previous paper (Otsuki et al. 2013) suggested, for small infants less than 7 kg, FFP of 10 ml/kg was routinely transfused preoperatively for hemispheric surgeries. The total blood loss for hemispheric surgeries was 150–250 ml and total time of the surgeries was 5–6 h.

**Fig. 3:**
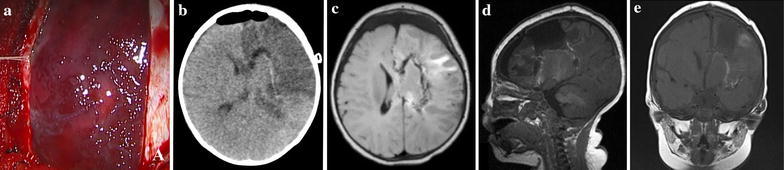
**a** Intraoperative image demonstrating leptomeningeal angioma; **b** Postoperative CT scan showing brain edema; **c**, **d**, **e** Postoperative MR images (axial, sagittal and cornonal view) confirming complete disconnection

The patient underwent a VPH to disconnect the entire cortex from the underlying diencephalic structures. A surgical specimen was sent to pathology and the histological examination of the resected material confirmed the diagnosis of SWS. She developed mild diabetes insipidus (DI) and edema in the left frontal lobe immediately after surgery, soon recovered and no replacement treatment was needed. Postoperative MR imaging demonstrated a complete disconnection of the affected hemisphere had been achieved (Fig. 3c–e).

The patient’s seizures ceased immediately after the surgery. At last follow-up, she has been free of seizures for more than 3 years with anti-epileptic drug (ZNS, 30 mg/day). She can walk without assistance and enjoy talking with her mother. Her right hand is auxiliary hand level and she can eat by herself using left hand and spoon. The patient’s developmental quotient (DQ) was improved from DQ 81 to DQ 85 (2014–2015).

## Discussion

This case demonstrates the utility of VPH as an effective surgical treatment for SWS patients with medically refractory epilepsy. VPH is an established treatment for intractable epilepsy due to diffuse hemispheric disease. Delalande (2007) reported the first series of 83 patients who underwent VPH for refractory epilepsy with shorter operative times and fewer early postoperative complications (Delalande et al. 2007). His surgical technique can achieve complete disconnection of the affected hemisphere and preserve an intact vessel supply (Delalande and Dorfmuller. 2008). However, the application of VPH to the SWS patients in early infancy is rarely reported. To our knowledge, only five cases younger than 1 year of age who underwent VPH have been previously reported (Delalande et al. 2007; Dorfer et al. 2013).

In SWS, 75 % of patients develop seizures during the first year of life. In addition, previous authors reported that early-onset seizures occurring in patients younger than 1 year of age may be more difficult to control and are associated with worse neurological and developmental outcomes (Alkonyi et al. 2011; Jagtap et al. 2013; Thomas et al. 2012). Numerous authors advocate extra-early hemispherotomy for hemispheric epileptic etiology including SWS because early-onset epilepsy that started in infancy always indicated a worse prognosis including medical intractability of the seizures, progressive hemiparesis, and mental retardation (Bourgeois et al. 2007; Tuxhorn and Pannek. 2002; Honda et al. 2013). It is worthy of note that, in younger children undergoing hemispheric surgery, significant intraoperative bleeding is common and is frequently seen in patients with malformations of cortical development which may be associated with hypovolemia and death (Bourgeois et al. 2007; Kossoff et al. 2002; Schramm et al. 2012). In a series of 27 patients with SWS, 3 required cerebrospinal fluid shunt placement, 6 had more than one operation because of residual lesion or the risk of massive hemorrhage (Bourgeois et al. 2007). The surgical techniques of VPH provide smaller skin incision and bone flap, which reduces blood loss and avoids the exposure of large venous sinuses. It allows complete disconnection of the hemisphere through a cortical window with good results in terms of seizure outcome and a relatively low complication rate (Delalande and Dorfmuller. 2008). We believe that this kind of disconnective technique can help reducing the potential complications associated with large brain excision (Delalande et al. 2007; Delalande and Dorfmuller 2008).

In the present case, VPH was performed in a 3-month-old infant with SWS and refractory seizures. Though PET showed some focal features, we performed hemispherotomy of VPH owing to the clinical hemispheric syndromes associated with a congenital hemispheric cerebral pathology. After surgery, she became seizure free and experienced improvement in her developmental status and motor performance that lasted up to her most recent follow-up (3 years after hemispherotomy). Our case concurs with those of many studies which have shown that hemispherectomy performed early in life is associated with minimal hemiparesis and better intellectual development (Table 1) (Bourgeois et al. 2007; Kossoff et al. 2002; Honda et al. 2013; Dorfer et al. 2013).

**Table 1:** Literature review of SWS surgery series in infancy

Authors and year	Age at Op (<1 year) (m)	Seizure duration (m)	Neurological deficit	Op	Seizure outcome	Developmental status
Ito et al. (1990)	5 and 11	3	Hemiparesis and psychomotor retarded	Parietooccipital lobectomy at 6 m and hemispherectomy at 11 m	None	Improved
	2	2	Poor head fixation	Hemispherectomy	None	Improved
Arzimanoglou et al. (2000)	8	6	Mild cognitive deficit	Hemispherectomy	None	Normal
Bourgeois et al. (2007)	6.3	4.4	Hemiparesis and severe development delay	hemispherectomy	None	Improved
	9.1	4.5	hemiplegia and severe development delay	Hemispherectomy	None	Improved
	5.7	0.8	Hemiparesis and moderate development delay	Temporoparietooccipital lobectomy	None	Improved
	8.5	5.1	Hemiparesis and severe development delay	Hemispherectomy	None	Unchanged
Delalande et al. (2007)	4	4	No details	Vertical parasagittal hemispherotomy	None	Improved
	12	6	No details	Vertical parasagittal hemispherotomy	None	Improved
	6	5	No details	Vertical parasagittal hemispherotomy	Partial seizure	Improved
Dorfer et al. (2013)	12	7	No details	Vertical parasagittal hemispherotomy	None	No details
	12	9	No details	Vertical parasagittal hemispherotomy	None	No details

Our patient developed transient DI and brain edema in the ipsilateral frontal lobe which produced mild midline shift. It is likely that the edema and DI was caused by the obstruction of venous drainage, but the underlying mechanism for such an association remains unknown. Previous data showed that manipulation and retraction of the fragile brain and vessels of infant may result in markable postoperative brain edema (Dorfer et al. 2015). We advocate that all infants should be extensively monitored for blood/volume imbalance, peripheral temperature and serum electrolytes. Based on our experience and literature reviews, we suggest that this kind of surgery should be performed in a pediatric epilepsy center where age-appropriate anesthesia and postoperative intensive care are available (Jagtap et al. 2013; Thomas et al. 2012; Bachur and Comi 2013; Alkonyi et al. 2011).

Jonas et al. reported that earlier surgery (<1 year) results in 76 % patients free of seizure compare to 58 % (age at operation >5 year) which demonstrated that surgery before the age of 1 year is favorable for good surgical outcome (Jonas et al. 2004). However, this data came from various etiologies, not solely on SWS. We suggest that VPH could reduce blood loss and avoid the exposure of large venus sinuses which should be a concern in infant surgery. Further, VPH exhibit good outcome of seizure control and improvement of hemiparesis in children with SWS and early-onset seizures. Future research in the form of more case reports and case series may add evidence to the literature about the use of VPH in the management of refractory epilepsy in infants with SWS.

## Conclusion

We have reported the VPH surgery on 3-month-old infant having an unilateral congenital nevus flammeus. VPH exhibit good outcome of seizure control and improvement of neurodevelopmental status and hemiparesis in children with SWS and early-onset seizures.
